# Valproate-associated hyperammonemic encephalopathy in subarachnoid hemorrhage: a diagnosis to consider

**DOI:** 10.5935/0103-507X.20220014-en

**Published:** 2022

**Authors:** Vivian Fuellis, Pedro Grille, Federico Verga, Luis Urbán Alfaro, Lucciano Grasiuso, Marcelo Barbato

**Affiliations:** 1 Intensive Care Unit, Hospital Maciel, ASSE - Montevideo, Uruguay.; 2 Service of Neurology, Hospital de Clínicas Doctor Manuel Quintela - Montevideo, Uruguay.

**Keywords:** Valproic acid, Anticonvulsants, Subarachnoid hemorrhage, Patient discharge, Brain injuries, Hyperammonemia, Morbidity

## Abstract

**Objective:** Subarachnoid hemorrhage is a prevalent disease with high morbidity and mortality. Numerous complications contribute to brain injury and defy the clinical practitioner on diagnosis and management. Valproate-associated hyperammonemic encephalopathy is a rare, underdiagnosed, serious and important entity to consider. We present a case of a patient with subarachnoid hemorrhage who received anticonvulsant prophylaxis with valproate and developed neuroworsening associated with high levels of ammoniemia and periodic discharge electroencephalographic patterns without other identifiable causes. Discontinuing valproic acid treatment and normalization of ammoniemia resulted in improvement in clinical and electroencephalographic neurological status.

## INTRODUCTION

Valproate (VPA) is a drug frequently used as an anticonvulsant and for psychiatric disorders. It is a short-chain fatty acid with hepatic metabolism. Disturbance of these hepatic processes may lead to an increase in serum ammonium.^(1)^ Valproate-associated hyperammonemic encephalopathy (VHE) is a rare, severe and reversible condition that is a consequence of an idiosyncratic reaction to acute or chronic treatment with VPA.^(2)^ Electroencephalography is useful in making the diagnosis and presents with a generalized periodic discharge (GPDs), a pattern often seen in toxic and/or metabolic encephalopathies.^(3)^

We analyzed the case of a patient with aneurysmal subarachnoid hemorrhage (SAH) receiving anticonvulsant prophylactic treatment with VPA who presented with impaired neurological status during hospitalization, in which a diagnosis of VHE was made. VPA toxicity mechanisms and pharmacokinetics, as well as clinical and laboratory aspects of VHE, are reviewed.

## CASE REPORT

A 69-year-old female, self-validating, with a history of hypertension, insulin-requiring diabetes, and smoking and a long history of headaches. She presented with a 1-week history of headache, confusion and vomiting.

**Figure 1:**
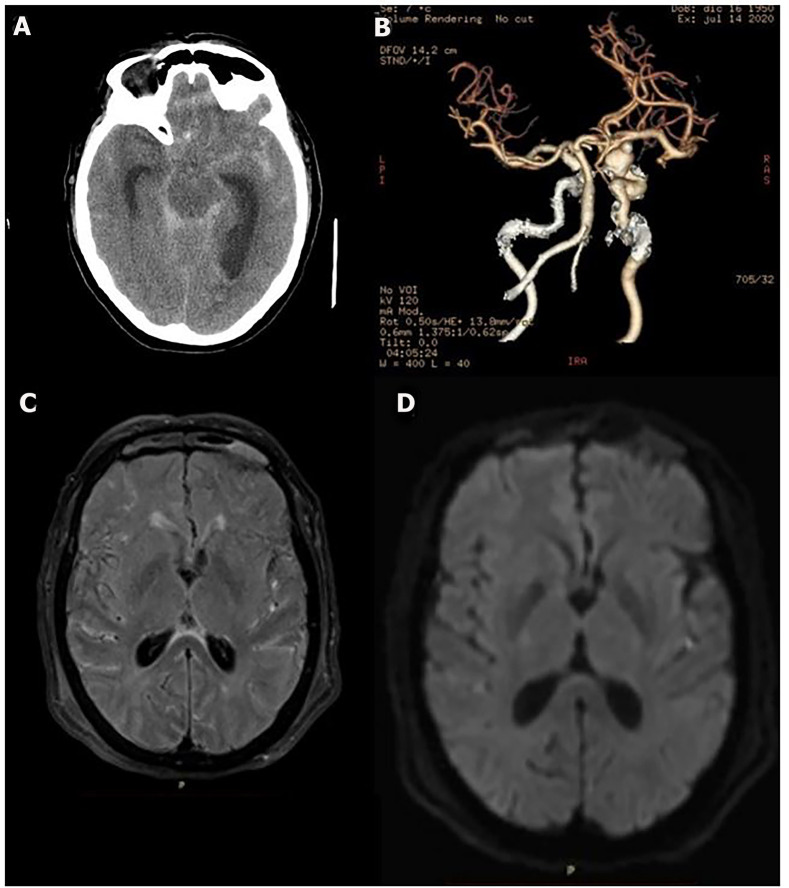
Neuroimaging studies of the case. (A) Head computed tomography scan showing diffuse subarachnoid hemorrhage with intraventricular hemorrhage and hydrocephalus; (B) computed tomography angiogram showing a right supraclinoid internal carotid artery aneurysm; (C) FLAIR magnetic resonance imaging and (D) diffusion magnetic resonance imaging show no abnormalities.

The examination at the emergency department revealed coma, i.e., a Glasgow coma scale score of 5, neck stiffness and no pupillary alterations. Computed tomography (CT) showed diffuse SAH, mild intraventricular hemorrhage and hydrocephalus (Figure 1A). A CT angiogram showed a right supraclinoid internal carotid artery aneurysm (Figure 1B). Once admitted to the intensive care unit (ICU), she received treatment with invasive mechanical ventilation, tranexamic acid, nimodipine and enteral VPA (400mg t.i.d.). External lumbar drainage was placed, and endovascular aneurysm coiling was performed without evidence of angiographic vasospasm. The initial CT showed hydrocephalus, so external ventricular drainage was placed.

As an initial complication, she developed neurocardiogenic dysfunction, with an *electrocardiogram* showing diffuse repolarization abnormalities and elevated troponins (716ng/mL). Transthoracic echocardiogram showed systolic dysfunction (*left ventricular ejection fraction*: 35%) and regional wall motion abnormalities, which were interpreted as stress cardiomyopathy. During the ICU stay, despite sedation withdrawal, the patient evolved to a coma (Glasgow coma scale score of 5 on Day 6). Subsequent CT scans were negative for hydrocephalus, ischemic lesions or other complications. Liver, renal and thyroid functions were normal, without hydroelectrolytic disorders. No extraneurological complications were found.

**Figure 2:**
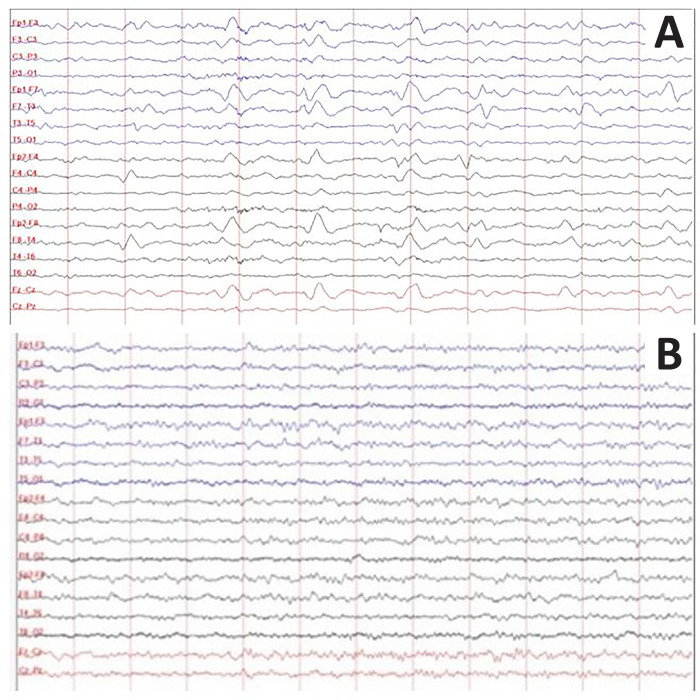
Electroencephalography of the case. (A) Standard encephalography on Day 6; (B) Standard encephalography on Day 12.

External ventricular drainage was removed with sterile cultures. Transcranial Doppler was negative for vasospasm and showed a normal pulsatility index. Magnetic resonance imaging (MRI) did not detect abnormalities (Figure 1C and 1D). Standard encephalography (EEG) ruled out ictal patterns but showed a GPD pattern with frontal predominance as a continuous periodic discharge pattern with triphasic morphology and no spontaneous fluctuation, as seen in toxic and/or metabolic brain dysfunctions (Figure 2A).^(4)^ Given these findings, the VPA dosage was 65.3µg/mL (therapeutic range: 50 - 100µg/mL), and the ammonium dosage was 160.2µg/mL (normal range: 18.7 - 86.0µg/mL). No hepatic failure or gastrointestinal bleeding was present. Then, VPA administration was discontinued, and treatment for hyperammonemia with lactulose and a protein-restricted diet was initiated. The ammonium level progressively decreased until normalization (81.7ug/mL), which was correlated with neurological improvements evidenced by a response to command at Day 12. Subsequent EEGs progressively improved until normal (Figure 2B).

The patient required tracheostomy to withdraw from mechanical ventilation and presented a tracheoesophageal fistula as a complication. She died 85 days after admission.

## DISCUSSION

Subarachnoid hemorrhage is a severe disease with a mortality up to 35 - 40% despite advances in treatment. It presents with multiple neurological and systemic complications that lead to secondary brain injury.

Valproate is a safe and effective drug used as an anticonvulsant prophylaxis in SAH patients. It increases cerebral GABA concentrations and produces a frequency-dependent blockage of voltage-dependent sodium channels that results in its anticonvulsant properties.^(1,2)^ Most of the side effects of VPA are mild, transient and related to dosage: dizziness, incoordination, diplopia and gait disturbance. Severe reactions such as hepatotoxicity, coagulation disorders, pancreatitis, teratogenicity, syndrome of inappropriate antidiuretic hormone secretion (SIADH), bone marrow aplasia, hyponatremia and encephalopathy are idiosyncratic.^(2)^

Valproate-associated hyperammonemic encephalopathy is a rare but severe complication linked to VPA treatment. It is characterized by a poor level of consciousness, neurological deficits, cognitive dysfunction, vomiting, drowsiness, lethargy and, in most severe cases, seizures, coma and death.^(2,3)^ Three clinical presentations have been described associated with the toxic effects of VPA: increased VPA serum levels with normal ammonia; encephalopathy with impaired liver function; and hyperammonemic encephalopathy. Our laboratory has an upper normal limit value of 86µg/mL for ammoniemia. Greater values result in toxicity, with 200µg/mL generating severe reactions such as cerebral edema and herniation.^(4)^ Our case is the first one described in our country and is the third clinical presentation of VHE.

There is no consensus on the mechanism by which VPA generates hyperammonemia. There are several theories, and the lack of investigation suggests a multifactorial process that involves genetic predisposition, drug interactions and multiple biochemical processes: indirect inhibition of fatty acid oxidation, which decreases N-acetyl-glutamate; direct effect of VPA on particular neurotransmitters, such as glutamine synthetase inhibition; and direct neuronal toxicity by an increase in intracellular astrocyte concentrations of glutamate and ammonium.^(3,4)^

There are several risk factors linked to the development of VHE: impaired liver function, carnitine deficiency, and associations with other drugs, such as phenytoin, phenobarbital, carbamazepine and topiramate. Other described risk factors, presented in our patient, include the recent initiation of VPA administration, high doses, hypercatabolic state and previous brain injury, the latter being probably the most relevant in our case. It is possible that toxic levels of VPA are related to a disruption in the blood-brain barrier as a result of SAH found in our patient, despite normal serum concentrations of VPA.^(1,2)^

Valproate-associated hyperammonemic encephalopathy incidence is not well documented. Up to 20% of patients under VPA treatment could develop mild, asymptomatic hyperammonemia. Another characteristic of this entity is the fact that no correlation between VPA concentrations and ammonium exists, so no relationship between dosage/ severity is found in VHE, which results in some patients presenting with normal VPA serum levels.^(1)^ It is necessary to have a high index of suspicion to make a correct diagnosis.

Encephalography is a tool used in noninvasive neuromonitoring that is performed at the bedside.

The following EEG patterns can be found in this entity: diffuse slowing of the background frequencies (with theta and delta waveform predominance); frontal, intermittent and rhythmic delta activity; or generalized periodic epileptiform discharges with anterior predominance and a triphasic morphology, as seen in our case.^(3,5,6)^ Since the first description of triphasic waves by Bickford et al., the diagnostic specificity of triphasic waves continues to be controversial, although most authors suggest that this pattern is more characteristic of hepatic encephalopathy.^(7)^ Fisch et al. found that triphasic waves occurring in hepatic encephalopathy were more likely to be associated with severe EEG slowing than other disorders, although that finding alone was not sufficient to distinguish from other causes of metabolic encephalopathies.^(8)^ Normalization of the EEG pattern after drug withdrawal is common and supports the diagnosis, as seen in our case.

There is no consensual treatment. The following steps are recommended: discontinue VPA treatment; treat triggering causes if there are any; enact measures to reduce ammonium levels (restriction of protein intake or use of nonabsorbable disaccharides such as lactulose); administering antibiotics such as rifaximin, neomycin and metronidazole for ammoniumproducing bacteria; administering levocarnitine, specifically during acute intoxication, given the interactions with VPA metabolism; and finally initiating hemodialysis or peritoneal dialysis when there is no response to the other treatments.^(1)^

The prognosis of VHE is good and without sequelae; as long as early diagnosis is made, the drug is discontinued, and the ammonium levels are normalized.

## CONCLUSION

Acute neurological impairment after subarachnoid hemorrhage is very frequent and is a diagnostic challenge for both clinicians and neurosurgeons. Once the most frequent causes of secondary brain injury have been excluded, valproate-associated hyperammonemic encephalopathy must be considered, since these patients present several risk factors, such as neurosurgery stress and acute illness. Knowing that valproate-associated hyperammonemic encephalopathy is a diagnosis of exclusion, a high index of suspicion is required along with the standard encephalography interpretation by the neurophysiologist and serum ammonium and valproate dosing. Prompt diagnosis and early discontinuation of the drug could change the course of this silent and life-threatening pathology.
